# Development, implementation and user experience of the Veterans Health Administration (VHA) dialysis dashboard

**DOI:** 10.1186/s12882-020-01798-6

**Published:** 2020-04-16

**Authors:** Michael J. Fischer, Wissam M. Kourany, Karen Sovern, Kurt Forrester, Cassandra Griffin, Nancy Lightner, Shawn Loftus, Katherine Murphy, Greg Roth, Paul M. Palevsky, Susan T. Crowley

**Affiliations:** 1 Jesse Brown VA Medical Center, Chicago, IL USA; 2 Center of Innovation for Complex Chronic Healthcare, Edward Hines Jr. VA Hospital, 5000 S. 5th Avenue (151H), Hines, IL 60141 USA; 3 University of Illinois Hospital and Health Sciences Center, Chicago, IL USA; 4 Veteran Affairs Durham Healthcare System, Durham, NC USA; 5 Duke University, Durham, NC USA; 6 Cincinnati VA Medical Center, Cincinnati, OH USA; 7 Department of Veterans Affairs, Washington DC, USA; 8 VA Connecticut Healthcare System, West Haven, CT USA; 9 Veterans Management Services, Inc., Indianapolis VA, Indianapolis, IN USA; 10 VA Pittsburgh Healthcare System, Pittsburgh, PA USA; 11 University of Pittsburgh School of Medicine, Pittsburgh, PA USA; 12 Yale University School of Medicine, New Haven, CT USA

**Keywords:** Hemodialysis, End-stage renal disease, Veteran, Performance measures, Implementation

## Abstract

**Background:**

Adults with end-stage renal disease (ESRD) requiring chronic dialysis continue to suffer from poor health outcomes and represent a population rightfully targeted for quality improvement. Electronic dashboards are increasingly used in healthcare to facilitate quality measurement and improvement. However, detailed descriptions of the creation of healthcare dashboards are uncommonly available and formal inquiry into perceptions, satisfaction, and utility by clinical users has been rarely conducted, particularly in the context of dialysis care. Therefore, we characterized the development, implementation and user experience with Veterans Health Administration (VHA) dialysis dashboard.

**Methods:**

A clinical-quality dialysis dashboard was implemented, which displays clinical performance measures (CPMs) for Veterans with ESRD receiving chronic hemodialysis at all VHA facilities. Data on user experience and perceptions were collected via an e-mail questionnaire to dialysis medical directors and nurse managers at these facilities.

**Results:**

Since 2016 the dialysis dashboard reports monthly on CPMs for approximately 3000 Veterans receiving chronic hemodialysis across 70 VHA dialysis facilities. Of 141 dialysis medical directors and nurse managers, 61 completed the questionnaire. Sixty-six percent of respondents did not find the dashboard difficult to access, 64% agreed that it is easy to use, 59% agreed that its layout is good, and the majority agreed that presentation of data is clear (54%), accurate (56%), and up-to-date (54%). Forty-eight percent of respondents indicated that it helped them improve patient care while 12% did not. Respondents indicated that they used the dialysis dashboard for clinical reporting (71%), quality assessment/performance improvement (QAPI) (62%), and decision-making (23%).

**Conclusions:**

Most users of the VHA dialysis dashboard found it accurate, up-to-date, easy to use, and helpful in improving patient care. It meets diverse user needs, including administrative reporting, clinical benchmarking and decision-making, and quality assurance and performance improvement (QAPI) activities. Moreover, the VHA dialysis dashboard affords national-, regional- and facility-level assessments of quality of care, guides and motivates best clinical practices, targets QAPI efforts, and informs and promotes population health management improvement efforts for Veterans receiving chronic hemodialysis.

## Background

The delivery of high quality and cost-effective chronic dialysis care for adults with end-stage renal disease (ESRD) remains a daunting challenge as evidenced by stagnant poor health outcomes and high costs across the United States (U.S.) [1, 2]. The prevalence of ESRD among Veterans is disproportionately high and approximately double that of non-Veterans, owing in part to high rates of predisposing comorbid illnesses, including diabetes mellitus, hypertension, older age and other sociodemographic risk factors [3]. Like their non-Veteran counterparts, Veterans receiving chronic hemodialysis experience an annual mortality rate greater than 15%, spend more than 30 days hospitalized annually, and have annual mean total healthcare costs approaching $140,000 [3, 4]. Such poor outcomes underscore the critical need to improve quality of chronic dialysis care.

Quality measurement for chronic dialysis care is well established in the U.S. Beginning in the 1990’s, the Centers for Medicare and Medicaid Services (CMS) required ESRD Networks to monitor and improve the quality of dialysis care. Over the past decade, CMS has iteratively developed and refined a variety of clinical performance measures (CPMs) for ESRD for public reporting (e.g., Dialysis Facility Compare) and value-based purchasing (e.g., Quality Incentive Program) using clinical practice guidelines as an initial framework, but extending beyond consensus guidelines. Although there is little formal evidence that use of quality measures improve healthcare quality [5], they have become an essential facet of the healthcare environment [6].

To further improve quality of healthcare, the U.S. government has prioritized and devoted substantial resources to promoting adoption of electronic health records and more broadly spurring on health information technology in medicine as illustrated by the Health Information Technology for Economic and Clinical Health Act in 2009 [7]. One such example is a healthcare dashboard, which is a data visualization tool designed to provide summary data on performance, as compared with standard metrics, in a clear, timely, and efficient manner [8, 9]. Healthcare dashboards have been increasingly adopted for a variety of purposes including decision support, monitoring quality and safety improvement efforts, optimizing clinical management systems, surveillance of adverse clinical events, financial reporting for clinical units, and for academic detailing of clinical specialists to remedy suboptimal performance [8–12].

Surprisingly, formal inquiry into the impact of dashboards on end-users in the healthcare environment has been rarely conducted [8]. While dashboards may facilitate improvements in adherence to clinical guidelines and safety measures, resource optimization and efficiency, and staff accountability and engagement, they may also entail unintended consequences such as greater staff workload, disruptions to clinical workflow, and improper focus and use of quality measures [8, 10, 11, 13–16]. Few studies have examined these potential tradeoffs by characterizing the creation, implementation, and use of healthcare dashboards [9–12, 14, 16, 17], and only two evaluated user perceptions and satisfaction [18, 19]. Moreover, although considerable heterogeneity exists in the clinical foci and settings of previously described dashboards, none focused on kidney disease [8]. Because of their unusually high costs and poor outcomes, adults with ESRD requiring chronic dialysis are a highly deserving clinical population for quality improvement [1], which could be facilitated by dashboard reporting.

The Veterans Health Administration (VHA) has been a leader in healthcare quality and leveraging quality measurement and health information technology [20–22]. In 2012, the VHA National Kidney Program began a process of leveraging multiple kidney disease and dialysis informatics initiatives to build an internal kidney disease surveillance system, which included the development of a VHA dialysis dashboard whose primary goal was to guide and motivate high quality care by providing national and facility level assessments of the quality of chronic dialysis care across VHA. In this manuscript, we report on the development, implementation, and user experience with the VHA dialysis dashboard.

## Methods

**Fig. 1:**
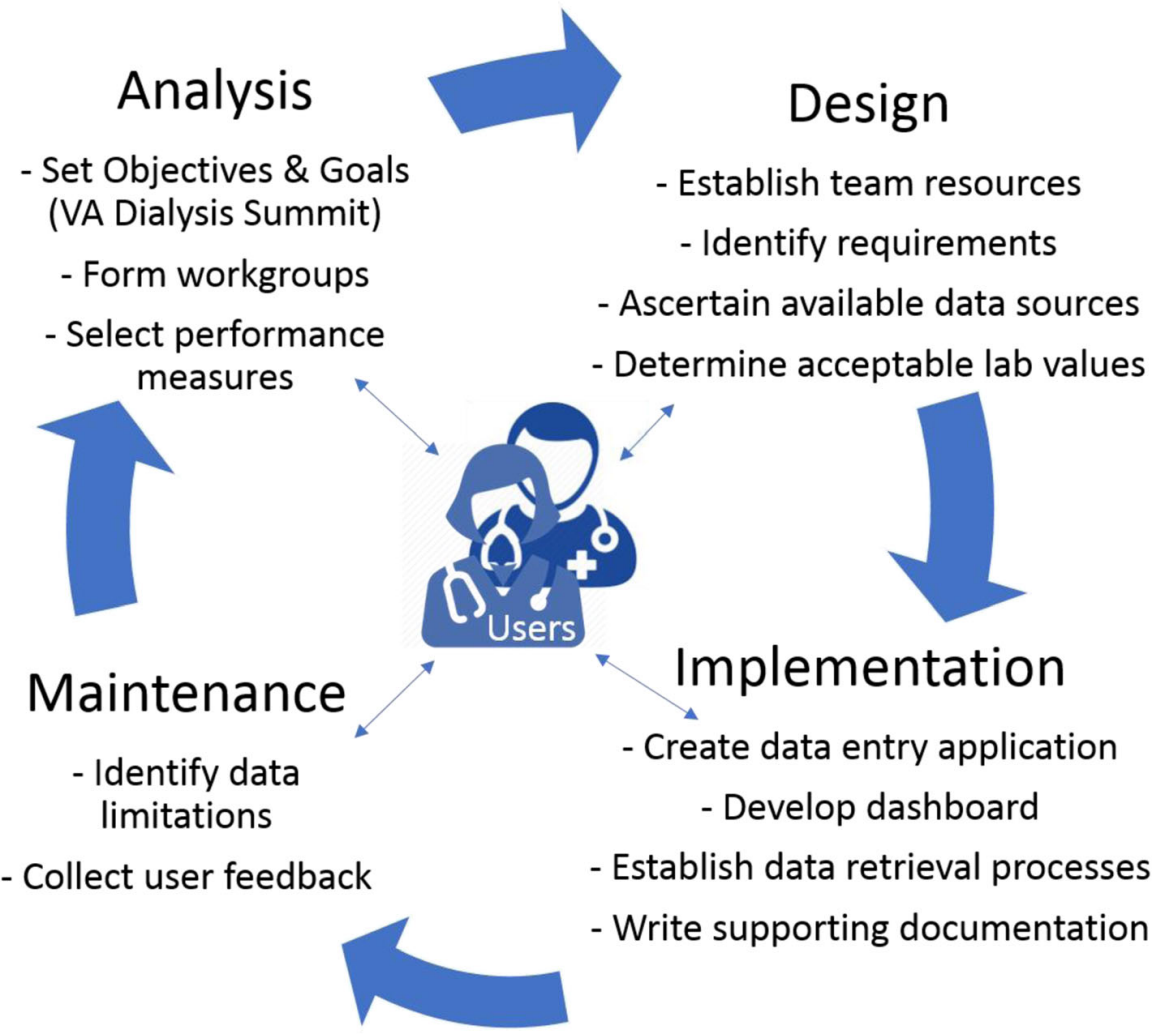
Development Process Used for the VHA Dialysis Dashboard

We employed a four-phase process to create a national VHA dialysis dashboard for capturing and reporting CPMs for approximately 3000 Veterans with ESRD receiving chronic hemodialysis at 70 VHA dialysis facilities across the U.S. (Fig. 1). Creation of the dialysis dashboard began in 2013 and by 2016 there was 100% dialysis facility participation. The primary expected user population consists of VHA nephrologists and dialysis staff (e.g., nurses, technicians).

### Analysis

The analysis phase involved establishing goals, forming a dashboard team, and selecting CPMs. There was broad consensus amongst VHA specialty care clinicians that the overarching goal was to report quality of VHA dialysis care through CPMs on an electronic dashboard that would be accessible to diverse stakeholders for patient care. A VHA dialysis dashboard team was assembled to serve as a steering committee. The team attended a dialysis summit meeting in November 2012 with representatives from various program offices that included the VHA National Program for Kidney Disease and Dialysis, VA Center for Applied System Engineering (VA CASE), VA Inpatient Evaluation Center (IPEC), VA Allocation Resource Center (ARC), VA Support Services Center (VSSC), VA Decision Support System (DSS), VA Patient Care Services (PCS), VA Health Services Research and Development (HSR&D), and VA Office of Information and Analytics (OIA). Early in this process, a CPM subcommittee with representation from leadership, operations, clinical services, and research was assembled to review, discuss, and select CPMs for this evaluation. The diversity of this stakeholder committee was deemed essential to achieve broad consensus for CPMs and ensure acceptance in the field.

Selecting ESRD CPMs for inclusion in the VHA dialysis dashboard consisted of an 8-month-long transparent, stakeholder-driven process. The first several meetings focused on establishing the principles to guide CPM selection. In consideration of known principles guiding selection criteria for performance measures, the CPM subcommittee sought to include measures that were timely, clinically relevant to chronic hemodialysis care, informed by solid evidence, and feasible and usable in VHA to enable robust improvement in the quality of VHA ESRD [23]. The subcommittee desired to have a mix of process and outcome measures that address different aspects of ESRD care [24]. Like other large healthcare reporting systems, the committee decided to use only explicit measures. Additionally, because the main purpose of the VHA dialysis dashboard is quality assessment and improvement at the facility level, only facility-level measures were chosen. Finally, because the initial focus of the VHA dialysis dashboard would be benchmarking to community performance, only mature vetted measures were considered.

The next several meetings of the performance measure subcommittee were focused on determining sources for CPMs. The subcommittee agreed to adopt CPMs from those already established and promulgated as mature measures by leading organizations instead of developing and vetting measures *de-novo* because these measures had already gained acceptance, would afford benchmarking, and lastly because of internal resource constraints. An environmental scan revealed five key sources for the potential ESRD CPMs: CMS, American Medical Association-Physician Consortium for Performance Improvement (AMA-PCPI), Kidney Care Quality Alliance (KCQA) and organizations that review, endorse, and adopt measures (National Quality Forum (NQF) and Ambulatory Care Quality Alliance (AQA). A total of 78 measures from these organizations were identified and they spanned broad ESRD clinical domains such as mortality, hospitalization, dialysis adequacy, vascular access, anemia, bone and mineral metabolism, infection, immunization, transplant, and end-of-life planning (Supplementary Table 1).

The last component of this process was a series of teleconference discussions and interval web-based voting and commenting by members of the subcommittee on these 78 CPMs, which last several months. Ultimately, 11 mature facility-level CPMs were selected out of the initial 78 measures identified from reference organizations. The final performance measures encompass dialysis solute clearance, vascular access, and surveillance measures of anemia management, bone and mineral metabolism, infection, and immunization (Table 1).

**Table 1:** VHA Dialysis Dashboard Clinical Performance Measures

Measure	Source	Type	Definition
Monthly measurement of delivered hemodialysis dose	CMS^a^	process	% patients w/ documented monthly adequacy measurements (spKt/V, URR)
Minimum delivered Hemodialysis dose	NQF^b^	Intermediate outcome	% patients on thrice-weekly hemodialysis whose average spKt/V > = 1.2 OR URR > 0.67
Minimizing use of catheters as chronic dialysis access	NQF	Intermediate outcome	% patients during the last HD treatment of month with a catheter continuously for 90 days or longer prior
Maximizing placement of arterial venous fistula (AVF)	NQF	Intermediate outcome	% patients during the last HD treatment of month using an autogenous AVF with two needles
Assessment of Iron Stores	CMS	process	% patients for whom serum ferritin and transferrin saturation percentage (TSAT) are measured simultaneously at least once during the rolling three-month study period.
Anemia Management: Hgb > 12	CMS	Intermediate outcome	% patients w/ hemoglobin value > 12.0 and on ESA
Measurement of serum phosphorus concentration	NQF	process	% patients with phosphorus measured at least once within month
Measurement of serum calcium concentration	CMS	process	% patients with calcium measured at least once within month
Proportion of patients with hypercalcemia	NQF	Intermediate outcome	% patients with 3-month rolling average of total uncorrected serum calcium > 10.2 mg/dL
Bloodstream infection measure	NQF	outcome	Number of outpatients with positive blood cultures per 100 hemodialysis patient-months
Influenza immunization	KCP^c^	process	% patients during the time from September 1 to March 31 who either received, were offered and declined, or were determined to have a medical contraindication to the influenza vaccine

^a^Centers for Medicare and Medicaid Services

^b^National Quality Forum

^c^Kidney Care Partners

### Design

Concurrently, the technical subcommittee investigated the availability of VHA data for the CPMs. They determined that gathering the extensive data needed would require not only leveraging the VHA electronic medical record and corporate data warehouse (CDW) but creating a web-based application for field reporting as well. Significant human resources and close collaboration across VA program offices and service lines were required to design a dashboard and were carried out through coordinated teleconferences over a 12-month period. The CPM subcommittee detailed specifications of the required data elements for each CPM, including acceptable ranges for laboratory values and proper calculation of derived values. For each selected CPM, the CPM subcommittee specified criteria for patient eligibility and inclusion/exclusion from numerator and denominator. A patient was eligible for inclusion in the CPM denominator if the patient had been receiving in-center outpatient hemodialysis treatments at the facility for at least 30 days as of the last day of the study period (e.g. calendar month). Secondary filters (e.g. ESRD duration, number of HD sessions in calendar month, etc.) were applied when applicable, in accordance with the technical specifications of the source where the measure was adopted from (e.g. NQF, CMS QIP, KCP). The subcommittee found that numerator and denominator specifications for established CPMs were often incomplete or insufficient for implementation in healthcare systems. Therefore, additional details were sought from available technical specification documents of measure developers, and/or reasonable standards were employed based on similar measures employed elsewhere. Specifications and eligibility naturally impacted CPM reporting and results. For example, for a few CPMs (e.g., minimum delivered hemodialysis dose), an additional requirement of ≥90 days since initiating maintenance HD was required for denominator inclusion. Also, some CPMs (e.g., monthly measurement of hemodialysis dose) required a patient to have ≥7 outpatient HD treatments at the dialysis facility during the calendar month for inclusion (in concurrence with CMS QIP criteria at that time), while others (e.g., maximizing AVFs) required ≥4 outpatient hemodialysis treatments during the calendar month for patient inclusion. The time period for numerator specifications also varied across CPMs. The majority required monthly assessment of whether patients met numerator criteria but a few such as the proportion of patients with hypercalcemia CPM required a 3-month rolling average assessment.

The technical subcommittee investigated the specific data elements needed for the CPMs and designed the general dashboard format, based on technology available in the VSSC. Analogous to the experience of others with national dashboards [10], significant variation in laboratory test names existed among VA facility laboratories across the United States. Although VHA is a national integrated healthcare system with a national data repository, laboratory test names are locally determined. To standardize the laboratory categories and facilitate extraction from VA electronic data warehouses, the dashboard team needed to correctly identify and map the laboratory names to standardized categories. Moreover, it is necessary for the team to continuously monitor local test names as new tests are made available within the system and program mapping must be updated to reflect these changes. We leveraged strategies and standards noted by others who have worked with VA data [25], including using the Logical Observation Identifiers Names and Codes (LOINC) framework to correctly structure and organize the data for abstraction. As part of the extraction of data elements from the various VA data sources methods for data mining as well as methods for synchronized nightly data extraction were specified via SQL queries. To supplement available elements extractable from exiting electronic databases, a web application was constructed to provide an interface for the dialysis clinical staff to report data that was otherwise unavailable.

The resultant web-based dashboard design was iteratively reviewed by human factors experts who applied usability heuristics and recommended format changes [26]. The teams made several key design decisions, including:

- □ numerical values achieve the goal of reporting CPM values better than charts [27]
- □ including a goal of ‘H’ (i.e., high) or “L’ (i.e., low) or a value where available, close to the actual values related to each measure allows for comparisons [28]
- □ presentation of historical values allows for local trend analysis [27]
- □ use of color
-  □ horizontal and vertical color shading separate CPM and historical groupings to reduce conjunction search time [29]
-  □ muted colors selected for their non-distracting appeal
-  □ not to use color to indicate CPM value proximity to goal because they would overwhelm end-users [30]
- □ linking to a file containing data definitions and to an email to the help desk if needed (help and documentation Heuristic and a VSSC standard)
- □ allowing export to other applications if needed (flexibility and efficiency of use Heuristic and a VSSC standard)

### Implementation

The implementation phase of VHA dialysis dashboard included pilot testing, a coordinated series of national rollout calls, and user acceptance testing. First, several VA dialysis facilities volunteered for pilot testing which involved validating eligible patient identification and extracted laboratory and medication data displayed on the dashboard. This process was critical in improving and refining the use of electronically abstracted data (e.g., administrative codes, laboratory values, etc.) for CPM calculation and patient identification. Identification of eligible patients from electronic records who met inclusion criteria was difficult because of issues such as patient vacation, interfacility transfers, and hospitalizations. Therefore, iterative modifications were required in selection algorithms with validation by these facilities. The pilot testing also allowed users to provide feedback regarding the web application for data entry. Second, a series of formal training sessions and rollout calls were held via teleconference with dialysis facility managers and the VHA dialysis dashboard team, which were critical for introducing the dashboard and receiving feedback. These sessions allowed for a detailed description of the functionality of the dashboard, including links to data definitions and patient level data, ways to export data for reports, and help desk links. Data security and dashboard access policies were reviewed at these sessions because of the importance to protect patient confidentiality. All stakeholders were reminded of VHA policies regarding patient identifying information (PII) and patient health information (PHI), including the required training for and regular review of all who access these data in conjunction with their job description and role in VHA. Moreover, access to the dashboard was subsequently enforced through network security measures. Finally, in collaboration with a usability lab, the VHA dialysis dashboard offered an extended period of user acceptance testing that was key to promoting an open forum for feedback and further validation from the field.

The final dashboard displays VHA’s dialysis facility performance on the CPMs in table form.

**Fig. 2:**
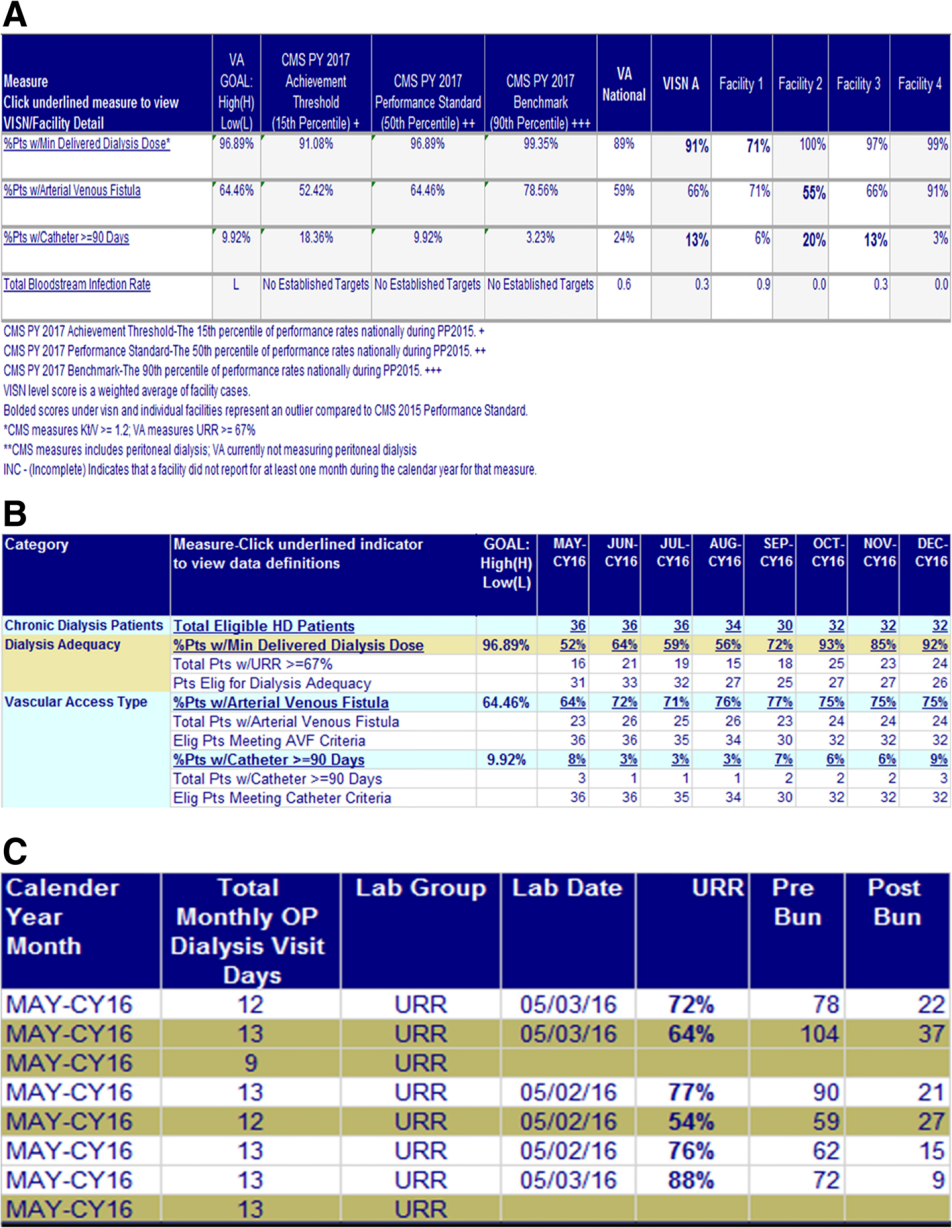
**a** VHA Dialysis Dashboard: National Level Data. **b** VHA Dialysis Dashboard: Facility Level Data. **c** VHA Dialysis Dashboard: Patient Level Data

It affords the user the ability to compare VHA national and facility performance with CMS benchmarks, other VHA facilities, and VHA national average (Fig. 2). It displays details of the measure (numerators and denominators) and provides comprehensive descriptions by selecting the measure. Furthermore, the user may drill down to patient level data for each CPM to explicitly determine the inputs for the performance calculation. Figure 2a shows a portion of the dashboard format for national summary data for each CPM while Fig. 2b shows the more detailed facility format and Fig. 2c show patient-level view. The user logon filters the visibility of facility and patient data.

### Maintenance

**Fig. 3:**
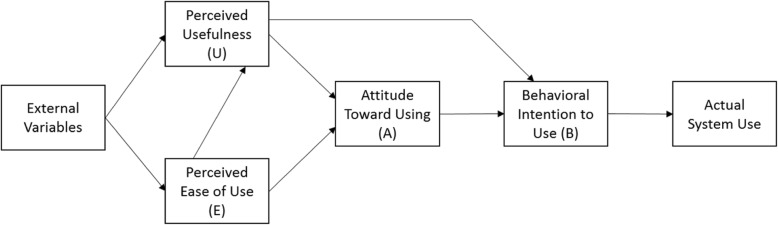
The Technology Acceptance Model

During the maintenance phase, while the field used the dashboard, the development team developed a questionnaire to evaluate the constructs of Perceived Ease of Use and Usefulness as defined in the Technology Acceptance Model (TAM) [31]. According to TAM, these constructs form the basis for actual system use (Fig. 3).

The questionnaire consisted of three parts: seven questions about demographics and professional experience, seven closed- and open-ended questions about use and application of the dashboard, and fifteen questions to assess perceptions of the dialysis dashboard on a Likert scale from one to five, representing ‘strongly disagree’ to ‘strongly agree’. A mix of negative and positive questions was used to reduce acquiescence bias [32]. Approximately 6 months after full implementation, an e-mail was sent to VHA nurse managers and medical directors at all 70 VHA outpatient dialysis clinics inviting them to participate in a confidential self-administered questionnaire (Additional file 3). This e-mail contained a short paragraph explaining the purpose of the questionnaire, which was to determine the role and usefulness of the VHA dialysis dashboard by assessing end-users’ use, satisfaction, and application of it to Veteran care. The survey was closed to end user respondents 6 weeks after the invitation distribution.

## Results

### Participant characteristics

Of the 141 VHA dialysis medical directors and nurse managers who were invited, 61 (43%) responded. Among the 61 respondents, 55 were aware of the VHA dialysis dashboard and had accessed it, 3 were aware of it but had not accessed it, and 3 were not aware of its availability. The final analytic cohort consisted of the 52 respondents who were aware of the VHA dialysis dashboard, had accessed it, and completed at least a portion of the questionnaire.

More than two-thirds of the respondents were 41 to 60 years of age (71%), female (69%), and nurse managers (73%) (Table 2). Nearly all respondents (88%) had been in their position for at least 1 year. Approximately one-half of respondents work with computers on their job greater than 6 h per work-day, and three-quarters (73%) characterize their computer skills and experience as average, and 65% characterize their skill and experience with medical information systems as average. A substantial majority of respondents (77%) use the VHA dialysis dashboard on a monthly or weekly basis.

**Table 2:** Characteristics of Respondents to VHA Dialysis Dashboard Survey (*n* = 52)

Characteristic	% (n)
Age (years)	
22–30	2 (1)
31–40	8 (4)
41–50	31 (16)
51–60	40 (21)
> 60	19 (10)
Sex	
Male	31 (16)
Female	69 (36)
Position	
Medical Director	19 (10)
Nurse Manager	73 (38)
Other	8 (4)
Duration in current position (years)	
< 1	12 (6)
1–5	42 (22)
6–10	19 (10)
> 10	27 (14)
Average frequency of dashboard use	
Daily	0 (0)
Weekly	12 (6)
Monthly	65 (34)
Quarterly	12 (6)
Annually	6 (3)
Other	6 (3)
Hours per day working with computers on job	
< 1	0 (0)
1–2	0 (0)
3–5	48 (25)
> 6	52 (27)
Skill and experience using computers	
basic	4 (2)
average	73 (38)
expert	23 (12)
Skill and experience using medical information systems	
basic	6 (3)
average	65 (34)
expert	29 (15)

### VHA Dialysis dashboard and clinical management

Dialysis staff indicated the predominant uses of the dialysis dashboard included clinical reporting (71%) and quality assessment and improvement (62%) (Table 3). They also used the dashboard data for clinical decision making (23%) and for comparisons with their own internal data and comparisons of their facility’s performance with other VA facilities. Sixty-five percent of respondents indicated that they used the dashboard with other applications including the Centers for Medicare and Medicaid Services (CMS) CROWNWeb national ESRD patient registry and quality measure reporting system, the VHA Medical Director toolkit, and National Kidney Foundation patient education tools.

**Table 3:** Uses of the VHA Dialysis Dashboard in Clinical Management (*n* = 52)

Types of Use	% (n)
**Clinical**	
Clinical reporting	71 (37)
Quality assessment and improvement	62 (32)
Clinical decision making	23 (12)
Other	15 (8)
**With other applications**	
Yes – CMS CROWNWeb patient registry, VHA Medical Director toolkit, National Kidney Foundation patient education tools	65 (34)
No	35 (18)

### Participant perceptions and experience with the VHA Dialysis dashboard

Sixty-six percent of respondents do not find the dashboard difficult to access and furthermore 64% agree that the dashboard is easy to use (Table 4). Approximately one-half of respondents agreed that the layout of the dialysis dashboard is good (59%) and that the presentation of data on the dialysis dashboard is clear (54%) and organized (48%), while few disagreed with these characterizations (≤ 20%). A substantial plurality of respondents did not find it difficult to find data on the dialysis dashboard (41%). Slightly more than 50% of respondents agreed that data for their facility on the dialysis dashboard is accurate and up-to-date. Few respondents agreed with the statement that the dialysis dashboard is not useful in taking care of dialysis patients (12%) while 48% indicated that it had helped them improve the care of dialysis patients. More respondents were satisfied (39%) than not satisfied (25%) with the dashboard’s design, and nearly 50% indicated that helpful answers to questions and support for the dashboard is sufficient. Additional written comments in space provided on the questionnaire included three respondents who stated that the dialysis dashboard is a ‘nice’ or ‘excellent tool’ and two respondents who desired more ‘up-to-date’ or ‘timely’ data on the dashboard. In subgroup analysis, responses among nurse managers/others and physician medical directors did not demonstrate substantial differences and revealed a high level of thematic convergence (Supplementary Table 2).

**Table 4:** Level of Agreement with Statements Regarding the VHA Dialysis Dashboard (*n* = 52)

**Statement**	**% (n) of responses**^**a**^					
**Perceived Ease of Use**	**1**	**2**	**3**	**4**	**5**	**None**
The VA Dialysis Dashboard is difficult to access	31 (16)	**34 (18)**	21 (11)	10 (5)	2 (1)	2 (1)
The VA Dialysis Dashboard is easy to use	2 (1)	12 (6)	21 (11)	**38 (20)**	25 (13)	2 (1)
The layout of the Dialysis Dashboard screen is good	0 (0)	19 (10)	19 (10)	**42 (22)**	17 (9)	2 (1)
It is difficult for me to find all the data that I am looking for on the VA Dialysis Dashboard	12 (6)	29 (15)	**27 (14)**	29 (15)	2 (1)	2 (1)
I have to CLICK too many times to find data on the Dialysis Dashboard	15 (8)	25 (13)	**27 (14)**	29 (15)	0 (0)	4 (2)
The presentation of data on the VA Dialysis Dashboard is clear	2 (1)	13 (7)	23 (12)	**46 (24)**	8 (4)	8 (4)
The presentation of data on the VA Dialysis Dashboard is well organized	2 (1)	12 (6)	31 (16)	**40 (21)**	8 (4)	8 (4)
I am satisfied with the design of the VA Dialysis Dashboard	2 (1)	23 (12)	**29 (15)**	35 (18)	4 (2)	8 (4)
**Statement**	**% (n) of responses**^**a**^					
**Perceived Usefulness**	**1**	**2**	**3**	**4**	**5**	**None**
The data for my facility on the VA Dialysis Dashboard is accurate	6 (3)	21 (11)	10 (5)	**52 (27)**	4 (2)	8 (4)
The data for my facility on the VA Dialysis Dashboard is up-to-date (current)	6 (3)	21 (11)	12 (6)	**50 (26)**	4 (2)	8 (4)
The VA Dialysis Dashboard is NOT useful to my job in taking care of VA dialysis patients	12 (6)	**42 (22)**	27 (14)	12 (6)	0 (0)	8 (4)
The VA Dialysis Dashboard has helped me to improve the care of dialysis patients	0 (0)	12 (6)	33 (17)	**42 (22)**	6 (3)	8 (4)
When I have questions about the VA Dialysis Dashboard, I am able to get prompt replies to my questions	4 (2)	10 (5)	31 (16)	**35 (18)**	15 (7)	8 (4)
When I have questions about the VA Dialysis Dashboard, I am able to get helpful answers to my questions	2 (1)	15 (7)	**31 (16)**	31 (16)	15 (7)	10 (5)
The VA Kidney Disease and Dialysis Program support for the Dialysis Dashboard is overall sufficient	2 (1)	12 (6)	27 (14)	**38 (20)**	12 (6)	10 (5)

^a^1 = Strongly disagree; 2 = Disagree; 3 = No opinion/Neutral; 4 = Agree; 5 = Strongly agree

Bolded cell indicates median response

### VHA Dialysis dashboard clinical performance measure results

During the 12-month period following full implementation of the VHA dialysis dashboard (i.e., the end of 2016 until the end of 2017), reporting of process measures (e.g., measurement of delivered hemodialysis dose, assessment of iron stores, measurement of serum calcium) on the dashboard revealed consistently high performance across all facilities with rather small performance gaps requiring improvement. In contrast, reporting of several intermediate outcome measures (e.g., placement of AVFs, use of chronic dialysis catheters) revealed a consistent performance gap across most facilities and a substantial variation in performance across facilities.

## Discussion

Since initial implementation, the VHA dialysis dashboard has reported monthly clinical performance for 11 quality measures for approximately 3000 Veterans at 70 VHA maintenance HD facilities across the United States. This electronic dashboard is accessible by VHA operations and clinical dialysis staff, and is being used for administrative reporting as well as quality assurance and performance improvement (QAPI) activities in important clinical areas of dialysis adequacy, vascular access, anemia, bone and mineral metabolism, and infection surveillance. Among VHA dialysis medical facility physician directors and nurse managers using the dialysis dashboard 1 year after its implementation with 100% facility participation, the majority found it accurate, up-to-date, easy to use, and helpful in improving the care of their patients. Nearly two-thirds of these users not only used the dialysis dashboard for clinical reporting and QAPI activities but also in combination with other clinical applications to enhance patient care.

As recently noted, healthcare organizations are increasingly implementing quality or clinical dashboards to improve patient care [8]. Similar to the electronic medical record, healthcare dashboards will continue to have a growing role in healthcare delivery, and they will need to be integrated by healthcare providers in their day-to-day care delivery. Nevertheless, only 14 published articles formally evaluated the impact of dashboards on clinical outcomes or examined clinician perceptions of dashboards and their utility [8–11]. Among these studies, 12 focused on single departments or facilities with relatively small sample sizes [8], and only one described a quality dashboard [18]. While clinical dashboards provide timely clinical data to clinicians so that they can make informed daily decisions about patient care, quality dashboards display information on performance measures at a unit or organizational level to assist administrators and managers with decision-making [8].

Although considerable heterogeneity exists in the clinical foci and settings of dashboards previous published studies, none focused on kidney disease [8]. Considering their increasing size, rising healthcare costs, and stagnating poor health outcomes, adults with ESRD requiring chronic dialysis are a highly deserving clinical population for quality improvement [1], which could be facilitated by dashboard reporting. The VHA dialysis dashboard combines features of both quality and clinical dashboards to drive improvement. It displays facility-level performance on quality measures with comparisons against recognized CMS benchmarks as well as other VHA facilities, and it is engineered with drill-down capability to patient-level clinical data. Hence, our report characterizing the creation and user experience of a clinical-quality dashboard used by dialysis facilities throughout a large national integrated healthcare system makes an important and timely contribution to literature.

Only two prior studies queried user satisfaction and perceptions of their dashboards [18, 19]. In three clinical areas of a National Health Service mental health trust in the United Kingdom, 21 mental health team members (nurses, medical staff, clerical staff) completed an anonymous questionnaire about their experiences with a recently implemented clinical-quality dashboard [18]. This dashboard used a variety of different graphics with color coding to present information ranging from clinical data such as number of available beds to quality measures such as percentage of fall assessments completed. Key findings included that 38% found the dashboard to be useful, 71% found it easy to use, and 86% found the format easy to understand. The most commonly cited benefits were timely access to information, increased communication and information-sharing, and data quality while difficulties included staff access, inaccurate data, and increased workload. In 2009, 175 clinical personnel (medical trainees, clerical staff, nurses, physicians) completed an anonymous written two-page survey regarding a recently implemented clinical dashboard in an emergency room in Beirut, Lebanon [19]. Key features of this dashboard included a patient severity index score as well as clinical laboratory and radiology results being laid out in a color-coded cubical form to designate whether the information had been reviewed by a team member. According to the few Likert scale questions that assessed user experience, 93% concurred (i.e., strongly agreed or agreed) that the dashboard was easy or extremely easy to use, 84% concurred that the dashboard allowed more time to take care of patients, and 83% concurred that the layout helped keep them organized [19]. In contrast, 38% of users felt that too many clicks were needed to navigate the dashboard.

The experience and satisfaction of nurse and physician users of the dialysis dashboard appears similar to that of these prior reports [18, 19]. Like prior studies, we designed our questionnaire to assess user perceptions around themes of access, ease of use, design, and clinical usefulness. In these domains, we noted similar findings since approximately 65% found the dialysis dashboard easy to use, less than 5% found it difficult to access or navigate, nearly 50% found that it helped to improve patient care, and a plurality if not a majority found the design clear, organized, and well laid out. We attribute some of these results to our extended period of user acceptance testing, feedback, and iterative refinement, which is well-known to be critical to successful usability [10, 33]. Recognizing their importance in sustained dashboard implementation [8], we also included questions to assess user perceptions of data integrity and support. Recognizing the reported wide variation in accuracy of electronically reported quality measures [34], we directed substantial efforts to data validation with the user field before dashboard implementation. Roughly 50% or more users of the dialysis dashboard viewed the data as timely and accurate and programmatic support for the dashboard as sufficient, while only 25% or less held an opposing viewpoint.

In contrast to prior studies, we also sought to understand how the dialysis dashboard was being used and incorporated by providers into patient care. Consistent with the objectives for creating the dialysis dashboard, nearly 2 two-thirds of physicians and nurse managers utilized the dashboard for clinical reports and QAPI activities. Moreover, users appear to be integrating the dashboard with other clinical applications such as existing CMS reporting portals and patient education materials for care delivery. However, the impact of the dialysis dashboard alone or in combination with other tools on processes of care and patient outcomes could not be assessed in this study. As noted elsewhere, there is scant evidence that clinical and quality dashboards have a positive effect on health outcomes, and more investigation is needed [8]. Moreover, quality dashboards may warrant scrutiny considering the recognized pitfalls and unintended consequences of quality measures, which include reporting burden, insufficient evidence, lack of validity, improper endorsement, inappropriate application, non-patient-centered, tunnel vision, measurement fixation, and misuse of healthcare resources [6, 35–39].

There are limitations to our study. Although we sampled all end users of the dialysis dashboard, our response rate was 43% and a higher proportion of nurse managers responded than physician medical directors; therefore, our findings may be subject to non-response bias. Because healthcare providers are generally more homogenous than the public in regard to knowledge, attitudes, and behavior, this concern is lessened [40]. Moreover, subgroup analyses comparing physician medical directors and nurse managers/others revealed a high level of thematic convergence in responses. Additionally, we purposely created a mix of questions where both high and low scores on Likert scales connoted a positive user response to reduce bias. Second, we assessed user perceptions and experience during a single time period. We recognize that such perceptions of the dashboard may change over time and impact sustainability. Lastly, while we surveyed users regarding how the dashboard was incorporated into clinical care, we had only a short period of observation (i.e., 12 months) to analyze changes in achievement of clinical performance measures coincident with questionnaire administration. Although the period of observation limits inferences of the effect of the dashboard on clinical practice and patient outcomes, these preliminary observations have already begun the VHA to focus efforts on best practices in regard to these clinical areas.

## Conclusions

The VHA clinical-quality dialysis dashboard affords national and facility level assessments of the quality of chronic dialysis care, guides and motivates best clinical practices, targets QAPI efforts at the facility and patient level, and promotes a cohesive approach to process improvement efforts at a national level for population management. The detailed multistep interdisciplinary stakeholder and user-driven implementation process for developing and implementing the dialysis dashboard serves as a prototype for dashboards of other chronic illnesses. Furthermore, characterizing the data selection and extraction processes for quality measurement and conveying an understanding of these methods is especially important since they have a large impact on the reliability of quality measure reporting, which is essential for the integrity of enterprise wide assessments and comparisons as well as stakeholder buy-in. While the dialysis dashboard offers tremendous potential to facilitate improvement in health outcomes for Veterans with ESRD requiring chronic dialysis, future work is needed to formally evaluate its impact on health outcomes.

## Supplementary information

**Additional file 1: Supplementary Table 1.** Initial Clinical Performance Measures Identified by the VHA Dialysis Dashboard Committee.

**Additional file 2: Supplementary Table 2.** Level of Agreement with Statements Regarding the VHA Dialysis Dashboard: Subgroup Findings.

**Additional file 3.** Survey of Dashboard Users – Medical Directors and Nurse Managers.

## Data Availability

The datasets generated and/or analyzed during the current activity are not publicly available due VHA policies, but are available from the corresponding author on reasonable request and if approved by VHA.

## Abbreviations

AQA: Ambulatory Care Quality Alliance
AMA-PCPI: American Medical Association-Physician Consortium for Performance Improvement
ARC: VA Allocation Resource Center
CDW: Corporate Data Warehouse
CMS: Centers for Medicare and Medicaid Services
CPMs: Clinical Performance Measures
DSS: VA Decision Support System
ESRD: End-Stage Renal Disease
HSR&D: VA Health Services Research and Development
IPEC: VA Inpatient Evaluation Center
KCQA: Kidney Care Quality Alliance
NQF: National Quality Forum
OIA: VA Office of Information and Analytics
PCS: VA Patient Care Services
QAPI: Quality Assurance and Performance Improvement
TAM: Technology Acceptance Model
U.S.: United States
VA CASE: VA Center for Applied System Engineering
VHA: Veterans Health Administration
VSSC: VA Support Services Center
