# Supplementary material for: Development, implementation and user experience of the Veterans Health Administration (VHA) dialysis dashboard
Source: BMC Nephrol. 2020 Apr 16;21:136. doi: 10.1186/s12882-020-01798-6 (PMC7160999; doi:10.1186/s12882-020-01798-6)
Supplement: Supplementary file 1 — Additional file 1: Supplementary Table 1. Initial Clinical Performance Measures Identified by the VHA Dialysis Dashboard Committee. [file 12882_2020_1798_MOESM1_ESM.docx]

**Supplementary Table 1: Initial Clinical Performance Measures Identified by the VHA Dialysis Dashboard Committee**

| **Measure** | **Source** | **Type** | **Definition** |
| --- | --- | --- | --- |
| Advance Directives Completed | AMA-PCPI^1^ | Process | Percentage of patients aged 18 years and older with a diagnosis of ESRD on hemodialysis or peritoneal dialysis who have advance directives or end of life medical orders completed based on their preferences |
| Referral to Hospice | AMA-PCPI | Process | Percentage of patients aged 18 years and older with a diagnosis of ESRD who withdraw from hemodialysis or peritoneal dialysis who are referred to hospice care |
| Influenza immunization | AMA-PCPI | process | Percentage of patients aged 6 months and older seen for a visit between October 1 and the end of February who received an influenza immunization OR patient reported previous receipt of an influenza immunization |
| Adequacy of Volume Management | AMA-PCPI | Intermediate outcome | Percentage of calendar months within a 12-month period during which patients aged 18 years and older with a diagnosis of ESRD undergoing maintenance hemodialysis in an outpatient dialysis facility have an assessment of the adequacy of volume management from a nephrologist |
| Influenza immunization | AMA-PCPI | process | Percentage of patients aged 6 months and older seen for a visit between October 1 and the end of February who received an influenza immunization OR patient reported previous receipt of an influenza immunization |
| ESRD Patients Receiving Dialysis: hemoglobin < 10 g/dL | AMA-PCPI | Intermediate outcome | Percentage of calendar months within a 12-month period during which patients aged 18 years and older with a diagnosis of ESRD who are receiving hemodialysis or peritoneal dialysis have a Hemoglobin level <10 g/dL |
| Patients on Erythropoiesis Stimulating Agent (ESA)--Hemoglobin Level >12.0 g/dL | AMA-PCPI | Intermediate outcome | Percentage of calendar months within a 12-month period during which a Hemoglobin is measured for patients aged 18 years and older with a diagnosis of advanced CKD (stage 4 or 5, not receiving RRT) or ESRD (who are on hemodialysis or peritoneal dialysis) who are also receiving ESA therapy have a Hemoglobin level > 12.0 g/dL |
| Arteriovenous Fistula Rate | AMA-PCPI | Intermediate outcome | Percentage of calendar months within a 12-month period during which patients aged 18 years and older with a diagnosis of ESRD and receiving maintenance hemodialysis are using an autogenous arteriovenous (AV) fistula with two needles |
| Catheter Use at Initiation of Hemodialysis | AMA-PCPI | Intermediate outcome | Percentage of patients aged 18 years and older with a diagnosis of ESRD who initiate maintenance hemodialysis during the measurement period, whose mode of vascular access is a catheter at the time maintenance hemodialysis is initiated |
| Catheter Use for ≥90 Days | AMA-PCPI | Intermediate outcome | Percentage of patients aged 18 years and older with a diagnosis of ESRD receiving maintenance hemodialysis for ≥ 90 days whose mode of vascular access is a catheter |
| Hemodialysis Adequacy: Solute | AMA-PCPI | Intermediate outcome | Percentage of calendar months within a 12-month period during which patients aged 18 years and older with a diagnosis of ESRD receiving hemodialysis three times a week for ≥ 90 days have a spKt/V ≥ 1.2 |
| Transplant Referral | AMA-PCPI | process | Percentage of patients aged 18 years and older with a diagnosis of ESRD on hemodialysis or peritoneal dialysis for 90 days or longer who are referred to a transplant center for kidney transplant evaluation within a 12-month period |
| Discussion of Advance Care Planning | AMA-PCPI | process | Percentage of patients aged 18 years and older with a diagnosis of ESRD on hemodialysis or peritoneal dialysis for whom there is documentation of a discussion regarding advance care planning |
| Influenza vaccination | AQA^2^ | process | Percentage of patients aged 18 years and older with a diagnosis of ESRD and receiving dialysis who received the influenza vaccination during the 12 month reporting period. |
| Plan of Care for Anemia | AQA | Intermediate outcome | Percentage of calendar months during the 12 month reporting period in which patients aged 18 years and older with a diagnosis of ESRD and receiving dialysis have a Hgb ≥ 11 AND patients have a Hgb < 11 with a documented plan of care. |
| Plan of Care for Inadequate Hemodialysis | AQA | Intermediate outcome | Percentage of calendar months during the 12 month reporting period in which patients aged 18 years and older with a diagnosis of ESRD and receiving hemodialysis have a Kt/V≥1.2 AND patients have a Kt/V<1.2 with a documented plan of care. |
| Vascular Access - Patients Receiving Hemodialysis | AQA | Intermediate outcome | Percentage of patients aged 18 years and older with a diagnosis of ESRD and receiving hemodialysis who have a functioning AV fistula AND patients who are referred for an AV fistula/permanent vascular access at least once during the 12 month reporting period. |
| Vascular Access - Patients Receiving Dialysis with a Permanent Catheter | AQA | Intermediate outcome | Percentage of patients aged 18 years and older with a diagnosis of ESRD with a permanent catheter after 90 days on dialysis who are referred for evaluation for permanent vascular access at least once during the 12 month reporting period. |
| Monthly measurement of delivered hemodialysis dose. | CMS^3^ | process | Percentage of all adult (≥ 18 years old) HD patients in the sample for analyses with documented monthly adequacy measurements (spKt/V) or its components in the calendar month |
| Measurement of Serum Calcium Concentration | CMS | process | Percentage of all adult (≥ 18 years of age) peritoneal dialysis and hemodialysis patients included in the sample for analysis with serum calcium measured at least once within month. |
| Anemia Management: Hgb > 12 | CMS | Intermediate outcome | This measure reports the percentage of eligible Medicare dialysis patients with a mean hemoglobin value greater than 12.0 g/dL. |
| Assessment of Iron Stores | CMS | process | Percentage of all adult (≥18 years old) hemodialysis or peritoneal dialysis patients prescribed an ESA at any time during the study period or who have a Hb <11.0 g/dL in at least one month of the study period for whom serum ferritin concentration AND either percent transferrin saturation or reticulocyte Hb content (CHr) are measured at least once during the study period for in-center hemodialysis patients, and at least twice during the study period for peritoneal dialysis patients and home hemodialysis patients.  peritoneal dialysis patients and home hemodialysis. |
| Facility Patient Survival Classification (based on SMR) | CMS | outcome | Risk-adjusted standardized mortality ratio for dialysis facility patients. |
| Dialysis adequacy: URR > = 65 | CMS | Intermediate outcome | This measure reports the percentage of eligible Medicare in-center hemodialysis (HD) patients with a median urea reduction ratio (URR) of at least 65%. |
| Measurement of Serum Phosphorus Concentration | CMS | process | Percentage of all adult (≥ 18 years of age) peritoneal dialysis and hemodialysis patients included in the sample for analysis with serum phosphorus measured at least once within month. |
| Hemoglobin Control for ESA Therapy | CMS | Intermediate outcome | Percentage of Adult (>= 18 years old) hemodialysis and peritoneal dialysis patients, with ESRD >= 3 months, who have received ESA therapy at any time during a 3 month study period AND who had Hb values reported for at least 2 of the 3 study months AND who achieved a mean hemoglobin of 10.0-12.0 g/dL for the 3 month study period. The hemoglobin value reported for the end of each reporting month (end-of- month Hemoglobin) is used for the calculation of the mean. |
| Monitoring Hemoglobin Levels Below Target Minimum | CMS | Intermediate outcome | Percentage of all adult (>=18 years old) hemodialysis or peritoneal dialysis patients with ESRD >=3 months and who had Hb values reported for at least 2 of the 3 study months, who have a mean Hb <10.0 g/dL for a 3 month study period, irrespective of ESA use. |
| Maximizing placement of AV Fistulae (AVF) | CMS | Intermediate outcome | Percentage of patients on maintenance hemodialysis during the last HD treatment of month using an autogenous AV fistula with two needles |
| Minimizing use of Catheters as Chronic Dialysis Access | CMS | Intermediate outcome | The percent of patients who are dialyzed with a chronic catheter (90 days or more) prior to the last hemodialysis session during the study period |
| Vascular Access Type: Catheter >= 90 days | CMS | Intermediate outcome | This measure reports the percentage of months where an intravenous catheter was in use for 90 days or more, among adult hemodialysis patients at the facility. |
| Vascular Access Type: Arterial Venous (AV) Fistula | CMS | intermediate outcome | This measure reports the percentage of months where an arterial venous (AV) fistula was in use, among adult hemodialysis patients at the facility. |
| Method of Measurement of Delivered Hemodialysis Dose. | CMS | process | Percentage of all adult (>= 18 years old) in-center HD patients in the sample for analyses for whom delivered HD dose was calculated using UKM or Daugirdas II during the study period and for whom the frequency of HD per week is specified. |
| Minimum Delivered Hemodialysis Dose for ESRD hemodialysis patients undergoing dialytic treatment for a period of 6 months or greater. | CMS | intermediate outcome | Percentage of all adult (≥ 18 years old) patients in the sample for analysis who have been on hemodialysis for 6 months or more and dialyzing thrice weekly whose average delivered dose of hemodialysis (calculated from the last measurements of the month using the UKM or Daugirdas II formula) was a spKt/V ≥ 1.2 during the study period. |
| Minimum Delivered Hemodialysis Dose for ESRD hemodialysis patients undergoing dialytic treatment for a period of 90 days or greater. | CMS | intermediate outcome | Percentage of all adult (>= 18 years old) patients in the sample for analysis who have been on hemodialysis for 90 days or more and dialyzing thrice weekly, and have a residual renal function (if measured in the last three months) less than 2 ml/min/1.73m2), whose delivered dose of hemodialysis (calculated from the last measurements of the month using the UKM or Daugirdas II formula) was a spKt/V >= 1.2 during the reporting period. |
| Percentage of the facility’s hemodialysis patients with a urea reduction ratio (URR) of 65% or greater in the calendar year | CMS | intermediate outcome | Eligible Medicare hemodialysis patients at the facility during the calendar year with a median URR value of 65% or higher |
| Percentage of Patients with Hemoglobin <10 g/dL | CMS | intermediate outcome | Hemodialysis and Peritoneal Dialysis patients, with ESRD >= 3 months, who have a mean Hemoglobin <10 g/dL for a 12 month reporting period, treated with ESA. The last valid hemoglobin value reported for the end of each reporting month (end-of-month Hemoglobin) is used for the calculation. |
| Percentage of Patients with Hemoglobin >12 g/dL | CMS | intermediate outcome | Hemodialysis and Peritoneal Dialysis patients, with ESRD > 3 months, who have a mean Hemoglobin >12 g/dL for a 12 month reporting period, treated with ESA. The last valid hemoglobin value reported for the end of each reporting month (end-of-month Hemoglobin) is used for the calculation. |
| Lower Limit for Serum Phosphorus | KCP^4^ | Intermediate outcome | Proportion of patients with 3-month rolling average of serum phosphorus less than 2.5 mg/dL |
| Use of Iron Therapy When Indicated | KCP | process | Percentage of all adult (>= 18 years old) dialysis patients with a serum ferritin < 100ng/mL and a TSAT < 50% on at least one simultaneous measurement who received IV iron in the following three months |
| Avoidance of Iron Therapy in Iron Overload | KCP | Intermediate outcome | Percentage of all adult (>= 18 years old) dialysis patients with a serum ferritin >= 1200 ng/mL or a TSAT >= 50% on at least one simultaneous measurement during the three-month study period who did not receive IV iron in the following three months. |
| Dietary Sodium Reduction Advice | KCP | Process | The proportion of patients who received formal advice on dietary sodium restriction by the renal dietician within the past 90 days. |
| Periodic Assessment of Post-Dialysis Weight by Nephrologists | KCP | Process | Proportion of patients who have documentation of receiving a new post-dialysis weight prescription from a nephrologist in the reporting month. |
| Utilization of High Ultrafiltration Rate for Fluid Removal | KCP | Process | The proportion of patients who did not receive an ultrafiltration (UF) rate greater than or equal to 15 mg/kg/hr in the reporting month. |
| Assessment of iron stores | KCP | Process | Percentage of all adult (>=18 years old) dialysis patients for whom serum ferritin and TSAT are measured simultaneously at least once during the three-month study period |
| Upper Limit for Total Uncorrected Serum Calcium | KCP | Intermediate outcome | Proportion of patients with 3-month rolling average of total uncorrected serum calcium greater than 10.2 mg/dL |
| IV Antibiotic Therapy (rate) | KCP | Process | Six-month rolling average rate of initiating IV antibiotic prescription therapy for newly suspected infection among adult chronic HD patients |
| Clinically Confirmed Infection *(rate)* | KCP | Intermediate outcome | Six-month rolling average rate of clinically confirmed infection with IV antibiotic therapy among adult chronic HD patients |
| Clinically Confirmed Infection *(percentage)* | KCP | Intermediate outcome | Six-month rolling average prevalence of clinically confirmed infection among HD patients prescribed IV antibiotics |
| Bacteremia (*rate*) | KCP | Intermediate outcome | Six-month rolling average rate of bacteremia with IV antibiotic therapy, among adult chronic HD patients |
| Bacteremia (*percentage*) | KCP | Intermediate outcome | Six-month rolling average prevalence of bacteremia among adult chronic HD patients prescribed IV antibiotics |
| VA-Related Infection *(rate)* | KCP | Intermediate outcome | Six-month rolling average rate of hemodialysis vascular access-related infection with IV antibiotic therapy and a clinically confirmed infection among adult chronic HD patients |
| VA-Related Infection *(percentage)* | KCP | Intermediate outcome | Six-month rolling average prevalence of hemodialysis access-related infection among adult chronic HD patients with a clinically confirmed infection and prescribed IV antibiotics |
| VA-Related Bacteremia *(percentage)* | KCP | Intermediate outcome | Six-month rolling average prevalence of bacteremia among adult chronic HD patients with a hemodialysis access-related infection and prescribed IV antibiotics |
| Catheter-Related Infection | KCP | Intermediate outcome | Six-month rolling average rate for access-related infection with IV antibiotic therapy, among adult chronic HD patients using a catheter for hemodialysis access |
| Catheter-Related Infection *(percentage)* | KCP | Intermediate outcome | Six-month rolling average prevalence of hemodialysis catheter-related infection among adult chronic HD patients with a HD access-related infection and prescribed IV antibiotics |
| Catheter-Related Bacteremia (*rate*) | KCP | Intermediate outcome | Six-month rolling average rate for access-related bacteremia with IV antibiotic therapy, among adult chronic HD patients using a catheter for hemodialysis access |
| Graft-Related Infection *(rate)* | KCP | Intermediate outcome | Six-month rolling average rate for access-related infection with IV antibiotic therapy, among adult chronic HD patients using an arteriovenous graft for hemodialysis access |
| Graft-Related Infection *(percentage)* | KCP | Intermediate outcome | Six-month rolling average prevalence of hemodialysis arteriovenous graft-related infection among adult chronic HD patients with a HD access-related infection and prescribed IV antibiotics . |
| Graft-Related Bacteremia (*rate*) | KCP | Intermediate outcome | Six-month rolling average rate for access-related bacteremia with IV antibiotic therapy, among adult chronic HD patients using an arteriovenous graft for hemodialysis access |
| Fistula-Related Infection *(rate)* | KCP | Intermediate outcome | Six-month rolling average rate for access-related infection with IV antibiotic therapy, among adult chronic HD patients using an arteriovenous fistula for hemodialysis access |
| Fistula-Related Infection *(percentage)* | KCP | Intermediate outcome | Six-month rolling average prevalence of hemodialysis arteriovenous fistula-related infection among adult chronic HD patients with a HD access-related infection and prescribed IV antibiotics |
| Fistula-Related Bacteremia (*rate*) | KCP | Intermediate outcome | Six-month rolling average rate for access-related bacteremia with IV antibiotic therapy, among adult chronic HD patients using an arteriovenous fistula for hemodialysis access |
| Unavailable Clinical Confirmation (*percentage*) | KCP | process | Six-month rolling average prevalence of “unavailable” information regarding clinical confirmation of infection among adult chronic HD patients with new IV antibiotic prescription |
| Unavailable Blood Culture Results (*percentage*) | KCP | process | Six-month rolling average prevalence of “unavailable” blood culture results for adult chronic HD patients with new IV antibiotic prescription |
| Sodium Profiling Practice for Hemodialysis | KCP | process | The proportion of hemodialysis patients who were not prescribed sodium profiling in the reporting month. |
| Restriction of Dialysate Sodium | KCP | process | The proportion of hemodialysis patients who were prescribed a dialysate sodium concentration less than or equal to 138 mEq/L in the reporting month. |
| Influenza Immunization in the ESRD Population (Facility Level) | KCQA^5^ | process | Percentage of end stage renal disease (ESRD) patients aged 6 months and older receiving hemodialysis or peritoneal dialysis during the time from October 1 (or when the influenza vaccine became available) to March 31 who either received, were offered and declined, or were determined to have a medical contraindication to the influenza vaccine. |
| Hemodialysis Adequacy-Minimum delivered Hemodialysis dose | NQF^6^ | Intermediate outcome | Percentage of all adult (>=18 years old) patients in the sample for analysis who have been on hemodialysis for 6 months or more and dialyzing thrice weekly whose average delivered dose of hemodialysis (calculated from the last measurements of the month using the UKM or Daugirdas II formula) was a spKt/V >= 1.2 during the study period. |
| Measurement of Serum phosphorus Concentration | NQF | process | Percentage of all adult (>= 18 years of age) peritoneal dialysis and hemodialysis patients included in the sample for analysis with serum phosphorus measured at least once within month. |
| Proportion of patients with hypercalcemia | NQF | Intermediate outcome | Proportion of patients with 3-month rolling average of total uncorrected serum calcium greater than 10.2 mg/dL. |
| Hemodialysis Vascular Access- Minimizing use of catheters as Chronic dialysis Access | NQF | Intermediate outcome | Percentage of patients on maintenance hemodialysis during the last HD treatment of study period with a chronic catheter continuously for 90 days or longer prior to the last hemodialysis session. |
| Hemodialysis Vascular Access- Maximizing placement of Arterial Venous Fistula (AVF) | NQF | Intermediate outcome | Percentage of patients on maintenance hemodialysis during the last HD treatment of month using an autogenous AV fistula with two needles. |
| National Healthcare Safety Network (NHSN) bloodstream infection measure | NQF | outcome | Number of hemodialysis outpatients with positive blood cultures per 100 hemodialysis patient-months. |
| Dialysis Facility risk-adjusted Standardized Mortality ratio | NQF | outcome | Risk-adjusted standardized mortality ratio for dialysis facility patients. |
| Patients on Erythropoiesis Stimulating Agent (ESA)—Hemoglobin level > 12.0 g/dl | NQF | Intermediate outcome | Percentage of calendar months within a 12-month period during which a Hemoglobin is measured for patients aged 18 years and older with a diagnosis of advanced CKD (stage 4 or 5, not receiving RRT) or ESRD (who are on hemodialysis or peritoneal dialysis) who are also receiving ESA therapy and have a Hemoglobin Level > 12.0 g/dL |
| Hemodialysis Adequacy: Solute | NQF | Intermediate outcome | Percentage of calendar months within a 12-month period during which patients aged 18 years and older with a diagnosis of ESRD receiving hemodialysis three times a week for ≥ 90 days have a spKt/V > or = 1.2. |
| Vascular Access—Functional AVF or AV Graft or Evaluation for placement | NQF | Intermediate outcome | Percentage of end stage renal disease (ESRD) patients aged 18 years and older receiving hemodialysis during the 12-month reporting period and on dialysis >90 days who:  1.have a functional autogenous AVF (defined as two needles used or a single-needle device [NOT one needle used in a two-needle device]) (computed and reported separately);  2.have a functional AV graft (computed and reported separately); or  3.have a catheter but have been seen/ evaluated by a vascular surgeon, other surgeon qualified in the area of vascular access, or interventional nephrologist trained in the primary placement of vascular access for a functional autogenous AVF or AV graft at least once during the 12-month reporting period (computed and reported separately). |
| Periodic assessment of post-dialysis weight by nephrologists | NQF | process | The proportion of in-center hemodialysis, home hemodialysis, and peritoneal dialysis patients who have documentation of receiving a new post-dialysis weight prescription from a nephrologist in the reporting month, irrespective of whether or not a change in post dialysis weight prescription was made |
| Standardized hospitalization ratio for admissions | NQF | outcome | Risk-adjusted standardized hospitalization ratio for admissions for dialysis facility patients. |

^1^American Medical Association-Physician Consortium for Performance Improvement

^2^Ambulatory Care Quality Alliance

^3^Centers for Medicare and Medicaid Services

^4^Kidney Care Partners

^5^Kidney Care Quality Alliance

^6^National Quality Forum
