# Supplementary material for: Development, implementation and user experience of the Veterans Health Administration (VHA) dialysis dashboard
Source: BMC Nephrol. 2020 Apr 16;21:136. doi: 10.1186/s12882-020-01798-6 (PMC7160999; doi:10.1186/s12882-020-01798-6)
Supplement: Supplementary file 2 — Additional file 2: Supplementary Table 2. Level of Agreement with Statements Regarding the VHA Dialysis Dashboard: Subgroup Findings. [file 12882_2020_1798_MOESM2_ESM.docx]

**Supplementary Table 2: Level of Agreement with Statements Regarding the VHA Dialysis Dashboard: Subgroup Findings**

| **Statement** | **% (n) of responses** | | | | | |
| --- | --- | --- | --- | --- | --- | --- |
| **Perceived Ease of Use** | **1** | **2** | **3** | **4** | **5** | **None** |
| The VA Dialysis Dashboard is difficult to access | 31 (16)  N/O: 31 (13)  **M: 30 (3)** | **34 (18)**  **N/O: 40 (17)**  M: 10 (1) | 21 (11)  N/O: 21 (9)  M: 20 (2) | 10 (5)  N/O: 7 (3)  M: 20 (2) | 2 (1)  N/O: 0 (0)  M: 10 (1) | 2 (1)  N/O: 0 (0)  M: 10 (1) |
| The VA Dialysis Dashboard is easy to use | 2 (1)  N/O: 0 (0)  M: 10 (1) | 12 (6)  N/O: 10 (4)  **M: 20 (2)** | 21 (11)  N/O: 21 (9)  **M: 20 (2)** | **38 (20)**  **N/O: 43 (18)**  **M: 20 (2)** | 25 (13)  N/O: 26 (11)  **M: 20 (2)** | 2 (1)  N/O: 0 (0)  M: 10 (1) |
| The layout of the Dialysis Dashboard screen is good | 0 (0)  N/O: 0 (0)  M: 0 (0) | 19 (10)  N/O: 21 (9)  M: 10 (1) | 19 (10)  N/O: 14 (6)  **M: 40 (4)** | **42 (22)**  **N/O: 45 (19)**  M: 30 (3) | 17 (9)  N/O: 19 (8)  M: 10 (1) | 2 (1)  N/O: 0 (0)  M: 10 (1) |
| It is difficult for me to find all the data that I am looking for on the VA Dialysis Dashboard | 12 (6)  N/O: 12 (5)  M: 10 (1) | 29 (15)  **N/O: 33 (14)**  M: 10 (1) | **27 (14)**  N/O: 29 (12)  M: 20 (2) | 29 (15)  N/O: 26 (11)  **M: 40 (4)** | 2 (1)  N/O: 0 (0)  M: 10 (1) | 2 (1)  N/O: 0 (0)  M: 10 (1) |
| I have to CLICK too many times to find data on the Dialysis Dashboard | 15 (8)  N/O: 17 (7)  M: 10 (1) | 25 (13)  N/O: 24 (10)  M: 30 (3) | **27 (14)**  **N/O: 31 (13)**  M: 10 (1) | 29 (15)  N/O: 26 (11)  **M: 40 (4)** | 0 (0)  N/O: 0 (0)  M: 0 (0) | 4 (2)  N/O: 2 (1)  M: 10 (1) |
| The presentation of data on the VA Dialysis Dashboard is clear | 2 (1)  N/O: 2 (1)  M: 0 (0) | 13 (7)  N/O: 14 (6)  M: 10 (1) | 23 (12)  N/O: 19 (8)  **M: 40 (4)** | **46 (24)**  **N/O: 50 (21)**  M: 30 (3) | 8 (4)  N/O: 7 (3)  M: 10 (1) | 8 (4)  N/O: 7 (3)  M: 10 (1) |
| The presentation of data on the VA Dialysis Dashboard is well organized | 2 (1)  N/O: 2 (1)  M: 0 (0) | 12 (6)  N/O: 12 (5)  M: 10 (1) | 31 (16)  N/O: 29 (12)  **M: 40 (4)** | **40 (21)**  **N/O: 43 (18)**  M: 30 (3) | 8 (4)  N/O: 7 (3)  M: 10 (1) | 8 (4)  N/O: 7 (3)  M: 10 (1) |
| I am satisfied with the design of the VA Dialysis Dashboard | 2 (1)  N/O: 2 (1)  M: 0 (0) | 23 (12)  N/O: 24 (10)  M: 20 (2) | **29 (15)**  N/O: 26 (11)  **M: 40 (4)** | 35 (18)  **N/O: 36 (15)**  M: 30 (3) | 4 (2)  N/O: 5 (2)  M: 0 (0) | 8 (4)  N/O: 7 (3)  M: 10 (1) |
| **Statement** | **% (n) of responses** | | | | | |
| **Perceived Usefulness** | **1** | **2** | **3** | **4** | **5** | **None** |
| The data for my facility on the VA Dialysis Dashboard is accurate | 6 (3)  N/O: 5 (2)  M: 10 (1) | 21 (11)  N/O: 21 (9)  M: 20 (2) | 10 (5)  N/O: 10 (4)  M: 10 (1) | **52 (27)**  **N/O: 55 (23)**  **M: 40 (4)** | 4 (2)  N/O: 2 (1)  M: 10 (1) | 8 (4)  N/O: 7 (3)  M: 10 (1) |
| The data for my facility on the VA Dialysis Dashboard is up-to-date (current) | 6 (3)  N/O: 2 (1)  M: 20 (2) | 21 (11)  N/O: 24 (10)  M: 10 (1) | 12 (6)  N/O: 12 (5)  M: 10 (1) | **50 (26)**  **N/O: 55 (23)**  **M: 30 (3)** | 4 (2)  N/O: 0 (0)  M: 20 (2) | 8 (4)  N/O: 7 (3)  M: 10 (1) |
| The VA Dialysis Dashboard is NOT useful to my job in taking care of VA dialysis  patients | 12 (6)  N/O: 12 (5)  M: 10 (1) | **42 (22)**  **N/O: 43 (18)**  **M: 40 (4)** | 27 (14)  N/O: 31 (13)  M: 10 (1) | 12 (6)  N/O: 7 (3)  M: 30 (3) | 0 (0)  N/O: 0 (0)  M: 0 (0) | 8 (4)  N/O: 7 (3)  M: 10 (1) |
| The VA Dialysis Dashboard has helped me to improve the care of dialysis patients | 0 (0)  N/O: 0 (0)  M: 0 (0) | 12 (6)  N/O: 7 (3)  **M: 30 (3)** | 33 (17)  N/O: 33 (14)  **M: 30 (3)** | **42 (22)**  **N/O: 45 (19)**  **M: 30 (3)** | 6 (3)  N/O: 5 (2)  M: 10 (1) | 8 (4)  N/O: 7 (3)  M: 10 (1) |
| When I have questions about the VA Dialysis Dashboard, I am able to get prompt replies to my questions | 4 (2)  N/O: 2 (1)  M: 10 (1) | 10 (5)  N/O: 10 (4)  M: 10 (1) | 31 (16)  N/O: 31(13)  **M: 30 (3)** | **35 (18)**  **N/O: 36 (15)**  **M: 30 (3)** | 15 (7)  N/O: 14 (6)  M: 10 (1) | 8 (4)  N/O: 7 (3)  M: 10 (1) |
| When I have questions about the VA Dialysis Dashboard, I am able to get helpful answers to my questions | 2 (1)  N/O: 0 (0)  M: 10 (1) | 15 (7)  N/O: 12 (5)  M: 20 (2) | **31 (16)**  N/O: 29 (12)  **M: 40 (4)** | 31 (16)  **N/O: 36 (15)**  M: 10 (1) | 15 (7)  N/O: 14 (6)  M: 10 (1) | 10 (5)  N/O: 10 (4)  M: 10 (1) |
| The VA Kidney Disease and Dialysis Program support for the Dialysis Dashboard is overall sufficient | 2 (1)  N/O: 0 (0)  M: 10 (1) | 12 (6)  N/O: 10 (4)  M: 20 (2) | 27 (14)  N/O: 29 (12)  M: 20 (2) | **38 (20)**  **N/O: 40 (17)**  **M: 30 (3)** | 12 (6)  N/O: 12 (5)  M: 10 (1) | 10 (5)  N/O: 10 (4)  M: 10 (1) |

*1=Strongly disagree; 2=Disagree; 3=No opinion/Neutral; 4=Agree; 5=Strongly agree

Bolded cell indicates median response

N/O – nurse manager and other; n=42

M – physician medical director; n=10
