# Supplementary material for: Development, implementation and user experience of the Veterans Health Administration (VHA) dialysis dashboard
Source: BMC Nephrol. 2020 Apr 16;21:136. doi: 10.1186/s12882-020-01798-6 (PMC7160999; doi:10.1186/s12882-020-01798-6)
Supplement: Supplementary file 3 — Additional file 3. Survey of Dashboard Users – Medical Directors and Nurse Managers. [file 12882_2020_1798_MOESM3_ESM.docx]

**Appendix 1: Survey of Dashboard Users – Medical Directors and Nurse Managers**

End-stage renal disease is a common, burdensome, and costly chronic condition among Veterans.  Assessment and improvement of the quality of chronic hemodialysis care for Veterans with end-stage renal disease is an important VHA goal.  To that end, a national VHA Dialysis Dashboard for capturing and reporting of consensus-endorsed clinical performance measures for all Veterans receiving chronic hemodialysis at VHA facilities was launched in 2013.

In order to determine the role and usefulness of the VHA Dialysis Dashboard, the VHA National Kidney Disease and Dialysis Program is now conducting a survey of the VHA Dialysis Staff to assess their use, satisfaction, and application of the Dialysis Dashboard to Veteran care. As a medical director or nurse manager of a VHA outpatient dialysis facility, you are requested to participate in this brief survey. This survey has been designed so that it can be completed within a few minutes. All information obtained from you in this survey will be treated as confidential.  No identifying information will be used in any report. Your responses will not impact your relationship with your VA facility or the U.S. Department of Veterans Affairs.

If you have any questions, please contact Karen Sovern at Karen.Sovern@va.gov.

Thank you!

*The following set of questions are about your use and experience with the VA Dialysis Dashboard*

1.Are you aware of the VA Dialysis Dashboard?

-yes

-no (go to question # 10)

2.Have you ever used the VA Dialysis Dashboard?

-yes (go to question # 4)

-no

3.If not, what are the reasons why you have not used the VA Dialysis Dashboard (check all that apply)? (After they answer this question, then go to question #10)

-someone else on the care team at my facility accesses the Dashboard

-the dashboard is difficult to access

-I don’t have time to access the dashboard

-I don’t think that the dashboard is relevant to my job

-Other:

4.If yes, On average, how often do you access the VA Dialysis Dashboard?

-daily

-weekly

-monthly

-quarterly

-annually

-other:

5.How have you used the data on the VA Dialysis Dashboard (check all that apply)?

-quality assessment and improvement

-clinical reporting (e.g., to patients, staff, VA facility leadership, etc.)

-clinical decision making

-other

6.Have you used other clinical tools or applications in conjunction with data on the VA Dialysis Dashboard for patient care?

-No (then go to question #8)

-Yes

7. Please describe the names of tools or applications that you have used in conjunction with the data on the VA Dialysis Dashboard for patient care and how you have used them ( e.g. clinical decision making, quality assessment and improvement, clinical reporting, etc.)

8.Please indicate your level of agreement with the following statements

|  | Strongly disagree | Disagree | No opinion/ Neutral | Agree | Strongly agree |
| --- | --- | --- | --- | --- | --- |
| The VA Dialysis Dashboard is difficult to access |  |  |  |  |  |
| The VA Dialysis Dashboard is easy to use |  |  |  |  |  |
| The layout of the Dialysis Dashboard screen is good |  |  |  |  |  |
| It is difficult for me to find all the data that I am looking for on the VA Dialysis Dashboard |  |  |  |  |  |
| I have to CLICK too many times to find data on the Dialysis Dashboard |  |  |  |  |  |
| The presentation of data on the VA Dialysis Dashboard is clear |  |  |  |  |  |
| The presentation of data on the VA Dialysis Dashboard is well organized |  |  |  |  |  |
| The data for my facility on the VA Dialysis Dashboard is accurate |  |  |  |  |  |
| The data for my facility on the VA Dialysis Dashboard is up-to-date (current) |  |  |  |  |  |
| The VA Dialysis Dashboard is NOT useful to my job in taking care of VA dialysis  patients |  |  |  |  |  |
| The VA Dialysis Dashboard has helped me to improve the care of dialysis patients |  |  |  |  |  |
| I am satisfied with the design of the VA Dialysis Dashboard |  |  |  |  |  |
| When I have questions about the VA Dialysis Dashboard, I am able to get prompt replies to my questions |  |  |  |  |  |
| When I have questions about the VA Dialysis Dashboard, I am able to get helpful answers to my questions |  |  |  |  |  |
| The VA Kidney Disease and Dialysis Program support for the Dialysis Dashboard is overall sufficient |  |  |  |  |  |

9.Do you have any other comments or suggestions about how to improve use of the VA Dialysis Dashboard?

*The following set of questions are about your professional position and experience in VA*

10.What is your position?

-medical director

-nurse manager

-other

11.How long have you been in your current position?

-< 1 year

-1-5 years

-6-10 years

-> 10 years

12.What is your sex?

-male

-female

13. What is your age (years)?

-22-30

-31-40

-41-50

-51-60

->60

14.How many hours per day do you work with computers at your job?

-<1 hour

-1-2 hours

-3-5 hours

-> 6hours

15. How would you characterize your skill and experience in using computers?

-basic

-average

-expert

16. How would you characterize your skill and experience in using medical information systems?

-basic

-average

-expert
